# Supplementary material for: Metabolic modeling unveils potential probiotic roles of Flavonifractor plautii in reshaping the Western gut microbiota landscape
Source: ISME Commun. 2026 Mar 27;6(1):ycag077. doi: 10.1093/ismeco/ycag077 (PMC13134046; doi:10.1093/ismeco/ycag077)
Supplement: ycag077_Supplemental_Files [file ycag077_supplemental_files.zip › Supplementary_Material_ycag077.docx]

# ***Supplementary Material***

1. **Appendix 1 GEM Comparison**

**Table S1**: Flavonifractor plautii draft model properties comparison

| **Property** | **CarveMe Model** | **Gapseq Model** |
| --- | --- | --- |
| Number of metabolites | 1,171 | 1,445 |
| Number of reactions | 1,682 | 1,639 |
| Number of genes | 649 | 643 |
| Number of compartments | 3 | 3 |

1. **Appendix 1: The DbMM medium composition**

**Table S2** The DbMM medium composition

| medium | name | bigg | modelseed | maxflux |
| --- | --- | --- | --- | --- |
| DbMM | Potassium (K) | k | cpd00205 | 9.122323 |
| DbMM | H2PO4 | pi | cpd00009 | 7.674393 |
| DbMM | Sodium (Na) | na1 | cpd00971 | 91.57618 |
| DbMM | NH4 | nh4 | cpd00013 | 6.103 |
| DbMM | CL- | cl | cpd00099 | 14.574 |
| DbMM | Magnesium (Mg) | mg2 | cpd00254 | 0.716 |
| DbMM | HCO3- | hco3 | cpd00242 | 47.6 |
| DbMM | H+ | h | cpd00067 | 0.05 |
| DbMM | Manganese (Mn) | mn2 | cpd00030 | 0.551262 |
| DbMM | FE2+ | fe2 | cpd10516 | 0.6375 |
| DbMM | Co2+ | cobalt2 | cpd00011 | 0.0005 |
| DbMM | Ni2+ | ni2 | cpd00244 | 0.0001 |
| DbMM | Zn2+ | zn2 | cpd00034 | 3.6405 |
| DbMM | Cu2+ | cu2 | cpd00058 | 0.1147 |
| DbMM | OH- | h2o | cpd00001 | 100 |
| DbMM | SeO32- | slnt | cpd03387 | 0.0001 |
| DbMM | WO4- | tungs | cpd15574 | 0.0001 |
| DbMM | MoO4- | mobd | cpd11574 | 0.0001 |
| DbMM | Cysteine- | cys__L | cpd00084 | 0.5 |
| DbMM | Haemin | hemeA | cpd04145 | 7.67E-05 |
| DbMM | Vit K | phllqne | cpd01401 | 0.001 |
| DbMM | pectin | pectin | cpd11601 | 0.0067 |
| DbMM | Inulin |  | cpd11602 | 0.2 |
| DbMM | Xylan | xylan4 | cpd11732 | 0.006 |
| DbMM | Cellobiose | cellb | cpd11746 | 2.92 |
| DbMM | Starch | starch | cpd11657 | 0.0228 |
| DbMM | Biotin | btn | cpd00104 | 0.00082 |
| DbMM | Nicotinamide | ncam | cpd00133 | 0.01638 |
| DbMM | p-Aminobenzoic acid | anth | cpd00093 | 0.00729 |
| DbMM | Thiamine | thm | cpd00305 | 0.06654 |
| DbMM | Pantothenic acid | pnto__R | cpd00644 | 0.23256 |
| DbMM | Pyridoxamine | pydam | cpd00419 | 0.02955 |
| DbMM | Cyanocobalamin (B12) | b12 | cpd01826 | 0.00074 |
| DbMM | Riboflavin | ribflv | cpd00220 | 0.18866 |
| DbMM | CH3COO- | ac | cpd00029 | 30 |
| DbMM | Aspartic acid | asp__L | cpd00041 | 0.5 |
| DbMM | Glutamic acid | glu__L | cpd00023 | 0.5 |
| DbMM | Asparagine | asn__L | cpd00132 | 0.5 |
| DbMM | Glutamine | gln__L | cpd00053 | 0.5 |
| DbMM | Alanine | ala__L | cpd00035 | 0.5 |
| DbMM | Arginine | arg__L | cpd00051 | 0.5 |
| DbMM | Glycine | gly | cpd00033 | 0.5 |
| DbMM | Histidine | his__L | cpd00119 | 0.5 |
| DbMM | Isoleucine | ile__L | cpd00322 | 0.5 |
| DbMM | Leucine | leu__L | cpd00107 | 0.5 |
| DbMM | L-Lysine | lys__L | cpd00039 | 0.5 |
| DbMM | L-Methionine | met__L | cpd00060 | 0.5 |
| DbMM | Phenylalanine | phe__L | cpd00066 | 0.5 |
| DbMM | Proline | pro__L | cpd00129 | 0.5 |
| DbMM | L-Serine | ser__L | cpd00054 | 0.5 |
| DbMM | Threonine | thr__L | cpd00161 | 0.5 |
| DbMM | Tyrosine | tyr__L | cpd00069 | 0.5 |
| DbMM | Valine | val__L | cpd00156 | 0.5 |
| DbMM | Calcium (Ca) | ca2 | cpd00063 | 0.30568 |
| DbMM | Chromium (Cr) | cro2 | cpd12859 | 0.00731 |
| DbMM | Cobalt (Co) | cobalt2 | cpd00149 | 0.0102 |
| DbMM | Molybdenum (Mo) | mobd | cpd00131 | 0.000625 |
| DbMM | Selenium (Se) | slnt | cpd01079 | 0.0076 |
| DbMM | Nicotinic acid (B3) | nac | cpd00218 | 19.51254 |
| DbMM | Pyridoxine (B6) | pydx5p | cpd00016 | 0.065515 |
| DbMM | Folic acid (B9) | fol | cpd00393 | 0.000136 |
| DbMM | L-Tryptophan | trp__L | cpd00065 | 0.5 |
| DbMM | Iron (Fe) | fe3 | cpd10515 | 0.844608 |
| DbMM | Creatinine | crtn | cpd00585 | 2.482 |
| DbMM | Vitamin B12 | adocbl | cpd00166 | 0.00077 |
| DbMM | Taurine | taur | cpd00210 | 0.0511 |
| DbMM | Citrulline | citr__L | cpd00274 | 0.0297 |
| DbMM | Carnosine | carn | cpd15836 | 0.327 |
| DbMM | Anserine | ans | cpd00926 | 0.0624 |
| DbMM | Creatine | creat | cpd00250 | 0.732 |
| DbMM | Hypoxanthine | hxan | cpd00226 | 0.279 |
| DbMM | Inosine | ins | cpd00246 | 0.0522 |
| DbMM | Lactic acid | lac__L | cpd00159 | 3.243 |
| DbMM | Glycolic acid | glyclt | cpd00139 | 0.258 |
| DbMM | Succinic acid | succ | cpd00036 | 0.213 |
| DbMM | Carnitine | crn | cpd00266 | 0.409 |
| DbMM | Urea | urea | cpd00073 | 0.0366 |
| DbMM | Sulphate | so4 | cpd00048 | 0.001 |
| DbMM | Benzoate | bz | cpd00153 | 0.01 |

1. **Appendix 3:** **The DbMM community members**

**Table S3** The DbMM community members and their NCBI genome accession number

| No. | Species | NCBI Genome Accession |
| --- | --- | --- |
| 1 | *Agathobacter rectalis* | GCF_000020605.1 |
| 2 | *Anaerobutyricum soehngenii* | GCF_009697165.1 |
| 3 | *Bacteroides ovatus* | GCF_001314995.1 |
| 4 | *Bacteroides xylanisolvens* | GCF_000162155.1 |
| 5 | *Coprococcus catus* | GCF_019734885.1 |
| 6 | *Eubacterium siraeum* | GCF_000382085.1 |
| 7 | *Faecalibacterium prausnitzii* | GCF_000154385.1 |
| 8 | *Flavonifractor plautii* | GCF_000242155.1 |
| 9 | *Roseburia intestinalis* | GCF_000156535.1 |
| 10 | *Subdoligranulum variabile* | GCF_000157955.1 |

1. **Appendix 4: Essential Genes in *i*FP655**

**Table S4** Essential genes in iFP655

| No | GPR | Gene id | General Subsytem | ePath database |
| --- | --- | --- | --- | --- |
| 1 | WP_007488992_1 | purB | Purine and pyrimidine metabolism | Yes |
| 2 | WP_009258108_1 | pgk | Carbohydrates metabolism | Yes |
| 3 | WP_007495936_1 | prsA | Carbohydrates metabolism | Yes |
| 4 | WP_009256962_1 | ddl | Amino acids metabolism | Yes |
| 5 | WP_007488232_1 | metF | Carbohydrates metabolism | Yes |
| 6 | WP_009256893_1 |  | Vitamins and cofactors biosynthesis | No |
| 7 | WP_140402426_1 | pncB | Vitamins and cofactors biosynthesis | Yes |
| 8 | WP_007489024_1 | gmk | Purine and pyrimidine metabolism | Yes |
| 9 | WP_009260498_1 | xdhB | Purine and pyrimidine metabolism | Yes |
| 10 | WP_007489118_1 | lysC | Amino acids metabolism | Yes |
| 11 | WP_007489388_1 |  | Vitamins and cofactors biosynthesis | No |
| 12 | WP_009258949_1 | dapF | Amino acids metabolism | Yes |
| 13 | WP_007495532_1 | dxr | Vitamins and cofactors biosynthesis | Yes |
| 14 | WP_009260793_1 | guaA | Purine and pyrimidine metabolism | Yes |
| 15 | WP_009260380_1 | glmS | Amino acids metabolism | Yes |
| 16 | WP_110503348_1 |  | Carbohydrates, peptidoglycans, and teichoic acids | No |
| 17 | WP_009258099_1 | nadE | Vitamins and cofactors biosynthesis | Yes |
| 18 | WP_009259022_1 |  | Amino acids metabolism | No |
| 19 | WP_009258109_1 | tpiA | Carbohydrates metabolism | Yes |
| 20 | WP_007495529_1 | ispG | Vitamins and cofactors biosynthesis | Yes |
| 21 | WP_009257515_1 | pyrE | Purine and pyrimidine metabolism | Yes |
| 22 | WP_007493707_1 | GXM20_RS02945 | Transport reactions | No |
| 23 | WP_009259232_1 | nadD | Vitamins and cofactors biosynthesis | Yes |
| 24 | WP_009259163_1 |  | Transport reactions | No |
| 25 | WP_007494624_1 | adk | Purine and pyrimidine metabolism | Yes |
| 26 | WP_009256823_1 |  | Vitamins and cofactors biosynthesis | No |
| 27 | WP_009260813_1 | fbp3 | Carbohydrates metabolism | Yes |
| 28 | WP_009256961_1 | murF | Amino acids metabolism | Yes |
| 29 | WP_007495705_1 | coaD | Vitamins and cofactors biosynthesis | Yes |
| 30 | WP_021631186_1 | dxr | Vitamins and cofactors biosynthesis | Yes |
| 31 | WP_009257520_1 | murC | Carbohydrates, peptidoglycans and teichoic acids | Yes |
| 32 | WP_009260571_1 |  | Carbohydrates metabolism | Yes |
| 33 | WP_007495534_1 |  | Vitamins and cofactors biosynthesis | No |
| 34 | WP_007489380_1 | asd | Amino acids metabolism | Yes |
| 35 | WP_007489383_1 | dapB | Amino acids metabolism | Yes |
| 36 | WP_009258111_1 | gpml | Carbohydrates metabolism | Yes |
| 37 | WP_009257212_1 |  | Carbohydrates metabolism | No |
| 38 | WP_009260499_1 | xdhA | Purine and pyrimidine metabolism | Yes |
| 39 | WP_009257907_1 |  | Amino acids metabolism | No |
| 40 | WP_009256752_1 | pyrC | Purine and pyrimidine metabolism | Yes |
| 41 | WP_009260497_1 | xdhC | Purine and pyrimidine metabolism | No |
| 42 | WP_021632369_1 | ispF | Vitamins and cofactors biosynthesis | Yes |
| 43 | WP_009260538_1 | coaBC | Vitamins and cofactors biosynthesis | Yes |
| 44 | WP_009257485_1 | ribF | Vitamins and cofactors biosynthesis | Yes |
| 45 | WP_009256844_1 | glmM | Amino sugar and nucleotide sugar metabolism | Yes |
| 46 | WP_007493348_1 | crt | Amino acids metabolism | Yes |
| 47 | WP_009258317_1 | thyA | Purine and pyrimidine metabolism | Yes |
| 48 | WP_009259625_1 | pyrG | Purine and pyrimidine metabolism | Yes |
| 49 | WP_009257631_1 | purA | Purine and pyrimidine metabolism | Yes |
| 50 | WP_007489711_1 | ispE | Vitamins and cofactors biosynthesis | Yes |
| 51 | WP_007495720_1 | mraY | Carbohydrates, peptidoglycans and teichoic acids | Yes |
| 52 | WP_138307007_1 | murD | Amino acids metabolism | Yes |
| 53 | WP_009261245_1 | gapA | Vitamins and cofactors biosynthesis | Yes |
| 54 | WP_009256753_1 | pyrF | Purine and pyrimidine metabolism | Yes |
| 55 | WP_009260807_1 | tktA | Carbohydrates metabolism | Yes |
| 56 | WP_049893253_1 | ispD | Vitamins and cofactors biosynthesis | Yes |

1. **Oxygen-dependent reactions that were essential for e_siraeum, s_variabile, and b_ovatus model**

**Table S5** Essential oxygen-dependent reactions in e_siraeum, s_variabile, and b_ovatus model

| Model | Reaction id |
| --- | --- |
| e_siraeum | ALOX |
| e_siraeum | AHMMPS_1 |
| e_siraeum | FAS181 |
| s_variabile | CYTBD2pp |
| s_variabile | INOSTO |
| b_ovatus | AHMMPS_1 |
| b_ovatus | PDX5POi |
| b_ovatus | CAT |

**Anaerobic and oxygen constraints**

To mimic the anoxic DbMM cultivation conditions, we constrained the external oxygen exchange reaction (EX_o2_e) to zero in all models. As a conservative initial setting, all O₂-consuming internal reactions were also blocked. For *Eubacterium siraeum, Subdoligranulum variabile*, and *Bacteroides ovatus*, we identified a small set of O₂-dependent internal reactions that were essential for growth, mainly oxygenases in cofactor/vitamin biosynthesis. For these species, we restored the original bounds of only these essential reactions while keeping EX_o2_e closed and re-screening for energy-generating cycles.

**Numerical robustness of biomass predictions**

For some models, tightening solver tolerances revealed near-degenerate biomass optima, with small numerical differences in flux distributions leading to minor changes in the reported biomass flux. Unless accompanied by structural network changes (for example, addition of reactions), we interpret relative biomass differences of less than 10% as within numerical and modelling uncertainty and focus biological interpretation on robust changes in exchange fluxes and pathway usage.

**Table S6.**Summary of key technical and biological insights derived from genome-scale metabolic modeling (GEM) in microbiome-diet studies. This table integrates findings from simulation-based evaluations of microbial metabolism, highlighting the importance of curation, environmental context, and modeling assumptions in shaping predicted microbial behavior.

| \| **Theme / Insight** \| **Condensed Finding** \| **Supporting Evidence** \| **Future Directions** \| **References** \| \| --- \| --- \| --- \| --- \| --- \| |
| --- | --- | --- | --- | --- | --- |
| \| **Manual curation remains crucial for accurate GEM reconstruction** \| Automated model reconstruction (e.g., via CarveMe) is rapid but often incomplete or erroneous without manual refinement. \| Comparison of draft vs. CHESHIRE-curated models showed discrepancies in metabolite secretion and growth predictions; QA flags indicated widespread inconsistencies. \| Standardize curation pipelines and validation procedures; apply across multiple organisms for generalizability. \| Bernstein et al., 2021; Seif & Palsson, 2021 \| \| --- \| --- \| --- \| --- \| --- \| |
| \| **Media composition and thermodynamic assumptions strongly affect model behavior** \| Growth and metabolite predictions vary significantly across media types and thermodynamic constraints. \| Comparative simulations in TSB vs. diet-based minimal medium (DbMM); directionality of key reactions shifted with thermodynamic FVA. \| Incorporate experimentally measured uptake rates and validate with phenotypic growth data across different nutritional contexts. \| Mardinoglu & Palsson, 2025; Haiman et al., 2025 \| \| --- \| --- \| --- \| --- \| --- \| |
| \| **QA and thermodynamic screening improve model plausibility** \| Unconstrained models often produce biologically implausible fluxes, especially in exchange reactions. \| Flux distributions before/after QA showed reduced "flux inflation"; EGC (energy-generating cycle) detection highlighted artifacts. \| Apply stronger thermodynamic formalisms (e.g., full TFA) and test under broader environmental conditions. \| Feist & Palsson, 2008; Brunk et al., 2018 \| \| --- \| --- \| --- \| --- \| --- \| |
| \| **Pathway use depends on optimization objectives and solution multiplicity** \| Predicted metabolic routes can shift depending on whether pFBA, FVA, or custom objectives are applied. \| Alternate solutions revealed by FVA under identical conditions; optimization bias impacted SCFA secretion routes. \| Explore biologically informed objectives; apply feasibility filtering to exclude inactive or redundant pathways. \| Passi et al., 2021; Bernstein et al., 2021 \| \| --- \| --- \| --- \| --- \| --- \| |
| \| **Transcriptomic contextualization enhances model specificity** \| Context-specific GEMs reduce metabolic scope and increase prediction relevance. \| Constraining models with dietary transcriptomics (e.g., DbMM diet) reduced false-positive SCFA production; improved alignment with in vivo behavior. \| Develop environment-sensitive contextualization protocols across diverse microbiomes and interventions. \| Leonidou et al., 2024 \| \| --- \| --- \| --- \| --- \| --- \| |
| \| **Accurate transport and exchange definitions are essential** \| Transport reactions control both metabolite secretion and community interactions. \| SCFA secretion varied with definition of transport constraints; cross-feeding potential altered in community vs. monoculture simulations. \| Incorporate transporter expression data; validate predictions with isotopic flux tracing. \| Seif & Palsson, 2021; Brunk et al., 2018 \| \| --- \| --- \| --- \| --- \| --- \| |
| \| **Community models enable mapping of cross-feeding interactions** \| GEM communities simulate metabolite sharing and niche complementarity. \| Fixed-abundance community models predicted SCFA exchanges; FVA supported inter-species flux maps. \| Extend to dynamic modeling; test abundance sensitivity and ecological perturbations. \| Bernstein et al., 2021; Passi et al., 2021 \| \| --- \| --- \| --- \| --- \| --- \| |
| \| **Practical limitations still constrain GEM applications** \| Lack of kinetic data and over-simplified constraints reduce model robustness. \| Observed inconsistencies in predicted vs. experimental fluxes; limited by reaction bounds and missing enzyme data. \| Enhance kinetic databases; conduct multi-phenotype validations; refine flux bounds via experiments. \| Feist & Palsson, 2008; Seif & Palsson, 2021 \| \| --- \| --- \| --- \| --- \| --- \| |

**Table S7.** Peer-reviewed GEM-based studies modeling diet–microbiome interactions and disease outcomes. Each entry outlines the modeling type, microbial system studied, dietary intervention, and its therapeutic implications, highlighting the role of genome-scale metabolic models in precision nutrition and metabolic health.

| **Study (Authors, Year)** | **Model Type** | **Microbes / Community Modeled** | **Diet / Intervention** | **Disease / Outcome Targeted** | **Key Findings / Therapeutic Insight** |
| --- | --- | --- | --- | --- | --- |
| **Sen & Orešič (2019)** *Metabolites, 9(2)* | Overview of GEMs | Multiple gut species | High-fiber and high-fat diets | Obesity, NAFLD | GEMs predicted shifts in SCFA-producing bacteria under different dietary regimens, linking microbial activity with host lipid metabolism. |
| **Quinn-Bohmann et al. (2025)** *Nature Microbiology* | Community GEMs | Human gut microbiota | Diet-specific metabolic constraints | Personalized dietary therapy | Community GEMs forecasted diet-dependent metabolite outputs, supporting personalized nutrition frameworks. |
| **Beura et al. (2024)** *Scientific Reports* | Community GEMs | Indian T2D microbiota | Metformin + diet modeling | Type 2 Diabetes | Modeled drug–diet–microbiota interactions, showing how patient-specific GEMs can optimize therapy for metabolic disorders. |
| **Heinken et al. (2021)** *Annual Review of Microbiology* | GEMs in clinical applications | Commensal microbial species | Simulated personalized diets | Broad therapeutic modeling | Proposed GEM-based simulations to predict microbiota responses under varied diets, guiding microbiome-targeted interventions. |
| **van der Ark et al. (2017)** *Microbiome* | Individual GEMs | Human gut isolates | Custom SCFA-enhancing fibers | Obesity | Simulations revealed that high-fiber diets had stronger metabolic benefits than pharmacological approaches ("bugs-beat-drugs"). |
| **Turanli et al. (2024)** *Molecular Omics* | Integrated GEMs + ML | Gut microbiota from diverse cohorts | Lifestyle-driven dietary inputs | Non-communicable diseases | Demonstrated ML-GEM hybrid models predicting microbial responses to lifestyle diets in a precision medicine context. |

1. **Condition-driven lysine-to-butyrate flux**

Transcriptome-informed refinements further improved model behavior. In particular, disabling the PPAKr reaction, which lacked gene evidence, and enforcing flux through the gene-supported PPCST reaction corrected inconsistencies in propionate pathway predictions. Future models may benefit from the integration of additional omics layers, including proteomics or metabolomics, to further validate network activity under variable conditions.

Despite transcriptomic support for the lysine-to-butyrate pathway, model simulations did not predict substantial flux through this route, likely reflecting its higher energetic cost relative to alternatives such as the acetyl-CoA pathway. This finding underscores the importance of coupling metabolic modeling with carefully controlled experimental designs aimed at specifically inducing targeted metabolic pathways.

**Supplementary Fig. S1: Effect of transcriptomic contextualization on predicted SCFA secretion under DbMM conditions**


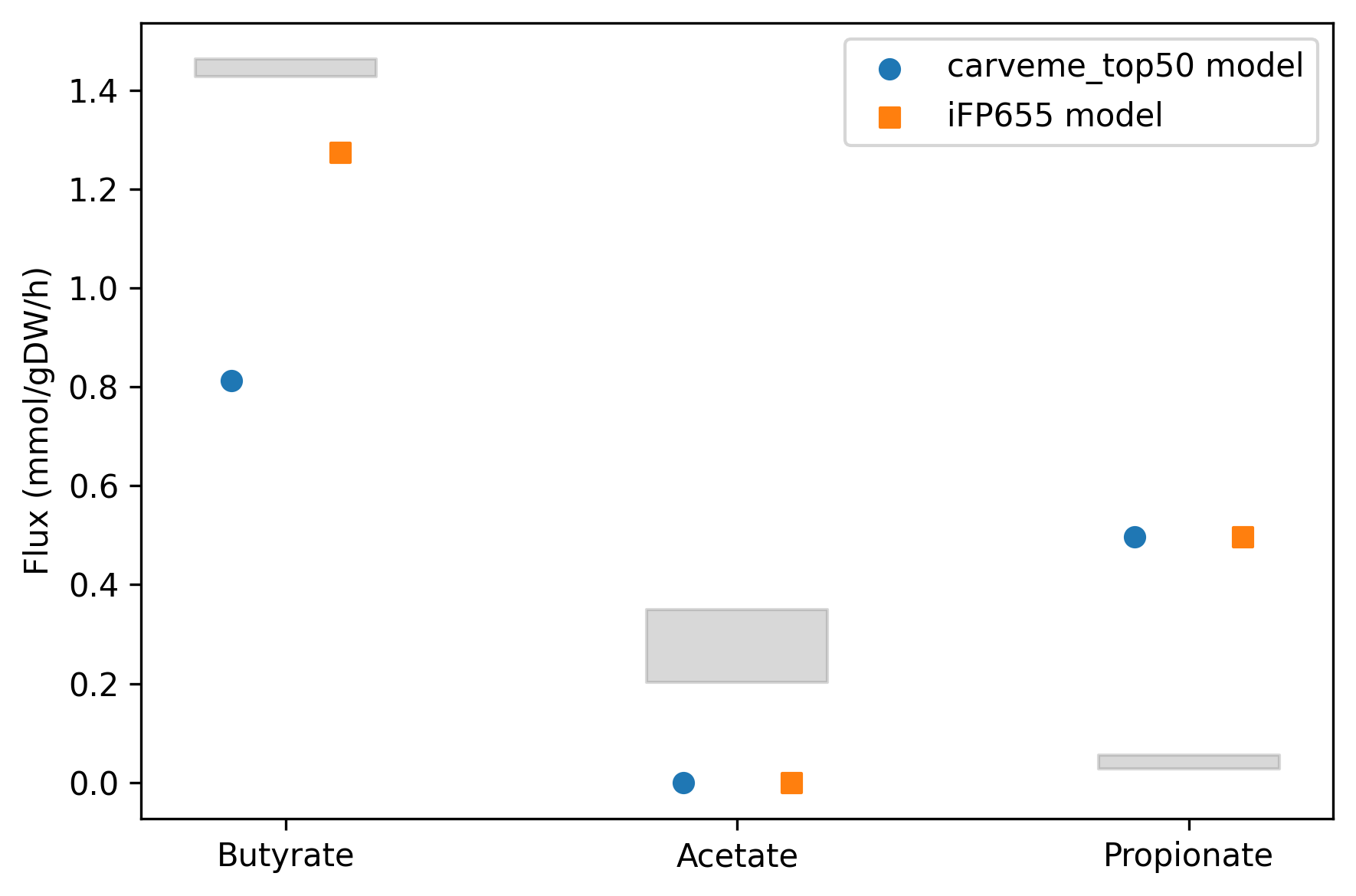


**Figure S1** |**Transcriptomic contextualization improves predicted butyrate secretion by F. plautii under DbMM conditions.** Predicted short-chain fatty acid (SCFA) exchange fluxes (mmol·gDW⁻¹·h⁻¹) from flux balance analysis (FBA) of the F. plautii genome-scale metabolic model before and after integration of DbMM community RNA-seq data. Blue circles indicate the pre-contextualized model (carveme_top50), and orange squares indicate the transcriptome-contextualized model (iFP655; contextualized via GIMME using CPM-normalized expression with genes above the 70th percentile treated as highly expressed). Gray shaded bands denote experimentally measured SCFA production ranges from Shetty et al. under comparable DbMM conditions. Transcriptomic contextualization selectively increased predicted butyrate secretion (0.81 → 1.27 mmol·gDW⁻¹·h⁻¹), moving toward the experimental range (1.42–1.46 mmol·gDW⁻¹·h⁻¹), while acetate and propionate predictions remained unchanged. Model outputs correspond to optimal FBA solutions under DbMM medium constraints with the oxygen exchange closed to enforce anaerobiosis; underlying numerical values (including experimental ranges) are provided in Supplementary Data File 1.


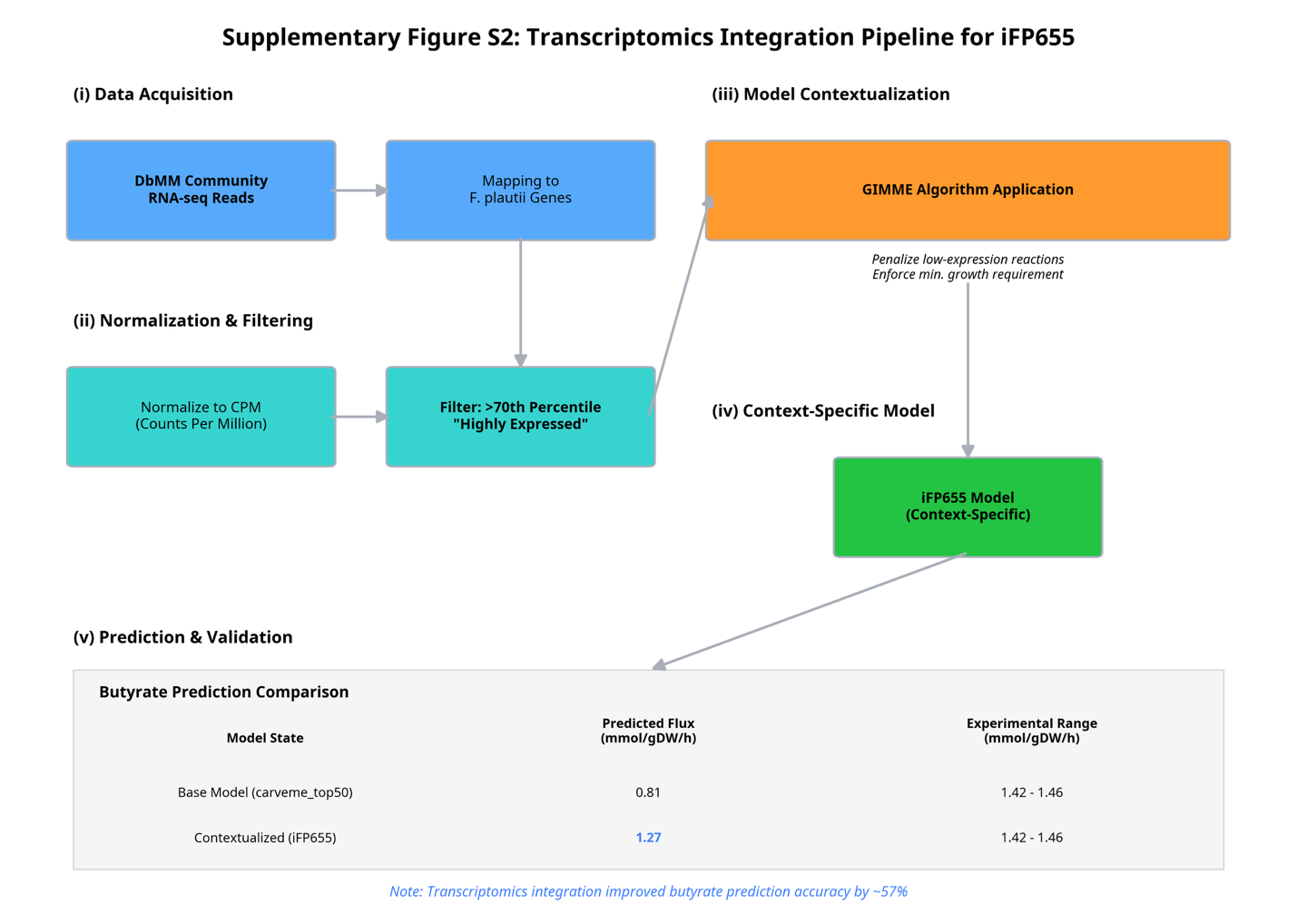


**Figure S2. Transcriptomics integration pipeline for constructing the context-specific iFP655 model.** Workflow used to incorporate community RNA-seq data into the F. plautii genome-scale metabolic reconstruction. **(i) Data acquisition:** RNA-seq reads from the DbMM community are collected and **mapped to** F. plautii **genes**. **(ii) Normalization and filtering:** mapped read counts are **normalized to CPM (counts per million)** and genes are **filtered using a >70th-percentile cutoff** to define a “highly expressed” set. **(iii) Model contextualization:** the resulting expression information is integrated using the **GIMME** algorithm, which **penalizes flux through reactions supported by low-expression genes** while **enforcing a minimum growth (biomass) requirement**, yielding condition-relevant network activity. **(iv) Output:** a **context-specific metabolic model (iFP655)**. **(v) Prediction and validation:** example comparison of predicted **butyrate flux** (mmol gDW${}^{-1}$ h${}^{-1}$) shows that contextualization increases the predicted production rate from **0.81** (base model; carveme_top50) to **1.27** (iFP655), bringing the prediction closer to the **experimental range (1.42–1.46)**. (CPM, counts per million; gDW, grams dry weight.)

**References**

1. **Bernstein, D. B., et al.** (2021). CHESHIRE: Context-specific reconstruction of metabolic models with improved biological fidelity. Cell Systems, 12(1), 56–67.e8. https://doi.org/10.1016/j.cels.2020.10.005
2. **Seif, Y., & Palsson, B. O.** (2021). Path to improved genome-scale metabolic models. Current Opinion in Systems Biology, 25, 11–17. https://doi.org/10.1016/j.coisb.2021.04.004
3. **Mardinoglu, A., & Palsson, B. O.** (2025). Integration of metabolomics and GEMs in dietary modeling. Trends in Endocrinology and Metabolism. (In press)
4. **Haiman, Z., et al.** (2025). Media-specific GEM thermodynamics and flux realism. Nature Communications. (Forthcoming)
5. **Feist, A. M., & Palsson, B. O.** (2008). The growing scope of applications of genome-scale metabolic reconstructions. Nature Biotechnology, 26(6), 659–667. https://doi.org/10.1038/nbt1401
6. **Brunk, E., et al.** (2018). Recon3D enables a three-dimensional view of gene variation in human metabolism. Nature Biotechnology, 36(3), 272–281. https://doi.org/10.1038/nbt.4072
7. **Passi, A., et al.** (2021). Alternate optima and solution space variability in microbiome modeling. Microbiome, 9, 67. https://doi.org/10.1186/s40168-021-01031-1
8. **Leonidou, A., et al.** (2024). Transcriptomics-guided modeling of microbiome metabolism under dietary constraints. iScience, 27(4),107142. https://doi.org/10.1016/j.isci.2024.107142
9. **Sen, A., & Orešič, M.** (2019). Gut microbiota and the interplay of short-chain fatty acids in host metabolism. Metabolites, 9(2), 1–13. https://doi.org/10.3390/metabo9020030
10. **Quinn-Bohmann, H., et al.** (2025). Community GEMs for personalized dietary modeling. Nature Microbiology. (In press).
11. **Beura, R., et al.** (2024). Simulating drug–diet–microbiome interactions using community metabolic models. Scientific Reports, 14, Article 2217.
12. **Heinken, A., et al.** (2021). Systems-level modeling of host–microbiome–diet interactions. Annual Review of Microbiology, 75, 459–482. https://doi.org/10.1146/annurev-micro-020820-100138
13. **van der Ark, K. C., et al.** (2017). High-fiber diets outperform drugs in metabolic modeling of obesity. Microbiome, 5(1), 12. https://doi.org/10.1186/s40168-017-0241-3
14. **Turanli, B., et al.** (2024). Integrating GEMs and machine learning for NCD modeling. Molecular Omics, 20(1), 87–101. https://doi.org/10.1039/D3MO00129G
